# One Health Approach to Rickettsiosis: A Five-Year Study on Spotted Fever Group Rickettsiae in Ticks Collected from Humans, Animals and Environment

**DOI:** 10.3390/microorganisms10010035

**Published:** 2021-12-25

**Authors:** Ilaria Pascucci, Elisa Antognini, Cristina Canonico, Marco Giuseppe Montalbano, Alessandro Necci, Alessandra di Donato, Martina Moriconi, Benedetto Morandi, Giulia Morganti, Silvia Crotti, Stefano Gavaudan

**Affiliations:** 1 Istituto Zooprofilattico Sperimentale dell’Umbria e delle Marche “Togo Rosati”, Via Gaetano Salvemini, 06126 Perugia, Italy; c.canonico@izsum.it (C.C.); Marcomontalbano1989@libero.it (M.G.M.); a.necci@izsum.it (A.N.); a.didonato@izsum.it (A.d.D.); m.moriconi@izsum.it (M.M.); b.morandi@izsum.it (B.M.); s.crotti@izsum.it (S.C.); s.gavaudan@izsum.it (S.G.); 2 Department of Veterinary Medicine, University of Perugia, Via San Costanzo 4, 06126 Perugia, Italy; giulia.morganti@unipg.it

**Keywords:** SFG Rickettsia, ticks, PCR, *Rickettsia rhipicephali*, *Rickettsia hoogstraalii*, Italy, human, animals

## Abstract

The spotted fever group of Rickettsiae is a heterogeneous group of Rickettsiae transmitted by ticks, causing similar diseases in humans (spotted fever). Until recently, it was supposed that a single pathogenic tick-borne SFG Rickettsia circulated in each different geographic area and that *R. conorii* subsp. *conorii* was the SFG Rickettsiae circulating in Italy, but in the last decade, thanks to molecular diagnostic, several different Rickettsia species, previously not considered pathogenic for decades, have been isolated from ticks and definitively associated to human disease, also in Italy. The present survey was carried out with the aim of investigating the presence of different SFG Rickettsia species in a geographic area where no information was available. Ticks collected from animals submitted to necropsy, removed from humans in local hospitals and collected from the environment were identified and tested by PCR for *Rickettsia* spp. based on the gltA gene, and positive PCR products were sequenced. A total of 3286 ticks were collected. Fifteen tick species were recognized, the most represented (79.52%) species in the collection was *Ixodes ricinus,* followed by *Rhipicephalus sanguineus* (9.13%). The overall prevalence of Rickettsia infection was 7.58%. Eight species of Rickettsia were identified, the most frequent was *R. monacensis* (56%), followed by *R. helvetica* (25.50%). Noteworthy, is the detection in the present study of *Rrhipicephali,* detected only twice in Italy. These are the first data available on SFG Rickettsiae circulation in the study area and they can be considered as starting point to assess the possible risk for humans.

## 1. Introduction

Rickettsioses are worldwide vector borne zoonoses caused by obligate intracellular Gram-native bacteria (order, *Rickettsiales*; family, *Rickettsiaceae*; genera *Rickettsia* and *Orientia*) infesting eukaryotic cells. They are among the oldest known vector-borne diseases [1]. Phylogenetics based on whole-genome analysis data determined that the genus *Rickettsia* comprises 27 recognized species and several dozen as-yet uncharacterized strains. According to Szokoli and collaborators [2], the *Rickettsia* genus could be divided into four groups: the spotted fever group (*R. rickettsii*, *R. conorii*, *R. parkeri*, and several others), typhus group (*R. prowazekii* and *R. typhi*), ancestral group (*R. bellii* and *R. canadensis*, not known to be pathogenic), and transitional group (*R. akari*, *R. australis*, and *R. felis*) [3]. The spotted fever group (SFG) is a heterogeneous group of Rickettsiae transmitted by ticks causing a similar disease in humans (spotted fever). Until recently, it was supposed that a single pathogenic tick-borne SFG Rickettsia circulated in each different geographic area, but in the last decade, to make puzzling the picture of SFG Rickettsiae in the world, several different *Rickettsia* species isolated from ticks, previously not considered pathogenic for decades, were definitively associated to human disease, as happened to *R. massiliae* [4]. Ticks are obligate hematophagous acarines that parasitize all vertebrates in almost every region of the world [5], they are second to mosquitoes as major vectors of pathogens to humans and play a primary role in disease transmission in animals [6]. However, given the transovarial transmission (TOT) with a high level of filial infection rate (FIR) described in ticks for the associated *Rickettsia* species, they are not only the vectors of SFG Rickettsiae, but they can be considered their main reservoir, even if for some SFG *Rickettsia* species, an amplifier role is suspected for some vertebrate species, such as rodents [4]. 

For this reason, SFG *Rickettsia* species and their respective vectors are very closely associated and, as consequence, the circulation of a SFG *Rickettsia* species in a territory strictly depends on the presence of its vectors and, consequently, is affected by the hosts’ population dynamics [7]. For many years, it was assumed that *R. conorii* subsp. *conorii* was the only agent of spotted fever circulating in Italy and that the pathognomonic clinical manifestation of Mediterranean spotted fever (MSF) was the only one developed after the SFG *Rickettsia* infection in humans. MSF, in fact, is constantly reported in warmer areas of the country during summer according to the biology of the tick *Rhipicepahlus sanguineus,* which, at those latitudes, behaves as an exophilic tick [1,4].

Thanks to the improved diagnostic skills and, mainly, to the use of molecular tools, in the last decade different SFG Rickettsiae have been identified in the Italian territory, including *Rickettsia slovaca*, *Rickettsia aeschlimanni*, *Rickettsia massilliae*, *Rickettsia monacensis*, *Rickettsia conorii* subsp. *Israelensis*, *Rickettsia conorii* subsp. *Indica*, *Rickettsia raoultii*, *Rickettsia helvetica*, *Rickettsia hoogstraalii*, *R. peacockii*, *R. rhiphicephali*, and *R. felis* [1,4,8,9,10,11,12,13].

Recently, in a retrospective study, 5989 cases of non-typhus rickettsioses were identified from 2001 to 2015 in humans in Italy, giving an average annual incidence of 0.88 (95%CI: 0.80; 0.96) per 100,000 [14]. In the Marche region, 72 human cases were reported in the same study and clinical cases are continuously reported in the region. As for other tick-borne diseases, a large part of the diagnosis of rickettsiosis in humans is made by clinical symptoms and serology, lacking the identification of the pathogen involved, and thus, the true incidence is likely to be underestimated, leading to the hypothesis that there are seven times more cases than officially reported [15].

The present study intended to investigate the circulation of SFG Rickettsiae in ticks collected in the Marche region and the bordering Republic of San Marino by specific molecular analysis and sequencing data, providing knowledge useful to better define the complex puzzle of SFG Rickettsiae circulation

## 2. Materials and Methods

### 2.1. Study Areas

**Figure 1:**
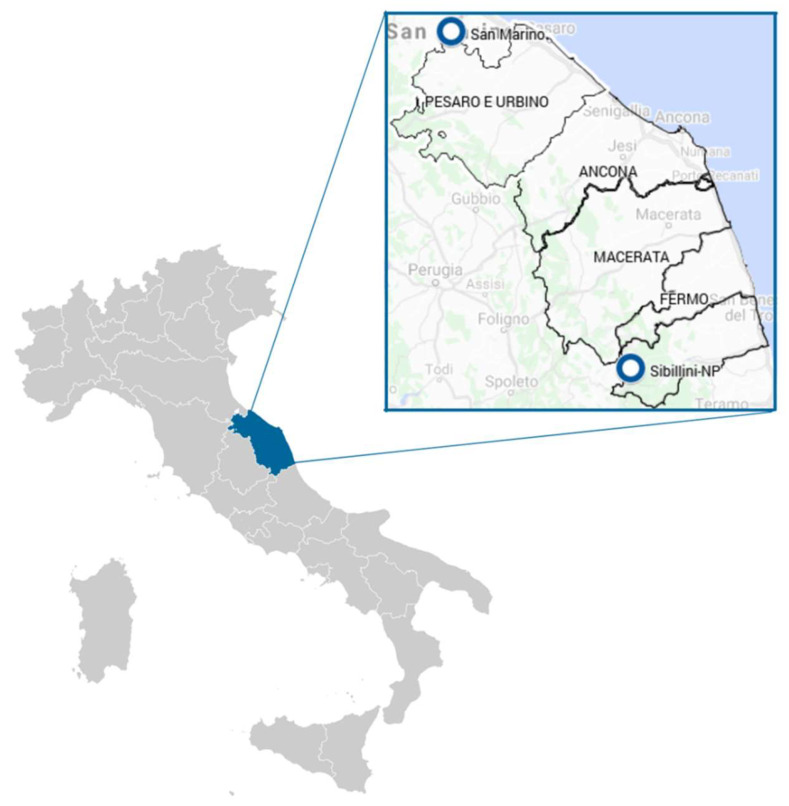
Geographical localization of the Marche region in Italy and of the dragging sites.

The Marche region is located in central Italy and extends over an area of 9694 square kilometers of the central Adriatic slope. Most of the region is mountainous or hilly, the main features being the Apennine chain along the internal boundary and an extensive system of hills rapidly descending towards the Adriatic. With the only exception of Monte Vettore (2476 m a.s.l.), the mountains do not exceed 2400 m a.s.l. The territory covers different environments characterized by high biodiversity in terms of wildlife, with several natural parks, such as Monti Sibillini National Park (Monti Sibillini NP) (Figure 1), a natural protected area of 71,437 square kilometers located in the Apenninic area of the region. Meanwhile, rural farming is common in the area, especially for small ruminants and beef cattle. Republic San Marino is an independent state landlocked by Italy, on the border between the regions of Emilia Romagna and Marche and about 10 km from the Adriatic coast (Figure 1). It is part of the Apennine mountain range and shares the same environmental features with the northern part of Marche region.

### 2.2. Sampling and Tick Identification

The Istituto Zooprofilattico Sperimentale dell’Umbria e delle Marche (IZSUM) is a veterinary public health laboratory the operates in the Umbria and Marche regions.

It receives carcasses of domestic and wild animals for diagnostic purposes and in the framework of specific animal health surveillance and control programs. Regarding zoonoses transmitted by ticks, in the Marche region, the Center for Entomology and Vector-borne Diseases (CERVe), located at IZSUM, has activated a service for tick identification and pathogen testing in ticks removed from humans.

In the period 2011–2015, ticks were collected from animal carcasses, humans, and the environment.

Ticks were collected from animals during the inspection before necropsy; carcasses were systematically examined for the presence of ticks around the soft parts of their bodies (ears, the inner sides of the hind and forelegs, perineal area), neck, and thorax.

Ticks recovered from humans were removed from patients by hospital medical staff and sent to IZSUM laboratory for species identification and pathogen detection. No clinical data of patients were available to the laboratory for privacy reasons.

Free-living ticks were collected in 22 sampling sites placed in two different geographical areas: 18 sites in the Republic of San Marino (RSM), and 4 sites in Monti Sibillini National Park (Figure 1). Dragging was performed during spring and fall using a 1 m^2^ white blanket, stopping every 2.5 m to prevent tick detachment. All ticks were immediately placed in flacons with 70% ethanol, properly labeled, and sent to IZSUM laboratory. Tick identification was performed using taxonomic keys available in the literature [16]. Before DNA extraction, immature free-living ticks were pooled according to species, dragging session details, and developmental stage, with a maximum of 100 larvae and of 50 nymphs in each pool, while adult ticks were tested singularly.

### 2.3. DNA Extraction and PCR

The single adult ticks and pooled samples were homogenized by TissueLyser (Qiagen) and DNA was extracted using the QIAamp^®^ cador^®^ Pathogen Mini Kit (Qiagen, Hilden, Germany), according to manufacturer’s protocol. Quality and quantity were tested with an Eppendorf biophotometer.

The extracted DNA was examined for the presence of *Rickettsia* spp. citrate synthase-encoding gene (gltA) by using RpCs877 F and RpCs1258 R primers (5′-GGGGGCCTGCTCACGGCGG-3′; 5′-AATTGCAAAAAGTACAGTGAACA-3′) and amplifying 381 base pair (bp) fragments as described by Roux and collaborators [17].

The reaction was performed in a total volume of 50 μL containing: 1× PCR buffer, 2.0 mM of MgCl_2_, 0.2 mM of dNTP, 0.5 μM of each primer, 0.05 U/μL of Taq DNA polymerase, 5.0 μL of template DNA, and distilled water. Positive and negative controls were included in each run. Cycling conditions for gltA amplification were: 5 min of denaturation at 94 °C, 35 cycles at 94 °C for 30 s, annealing for 45 s at 50 °C and 72 °C for 45 s, with a final extension step at 72 °C for 7 min. The amplifications were followed by horizontal electrophoresis on a 2% agarose gel in TAE 1× buffer (*w*/*v*), and were visualized with GelRed (10,000× Biotium) under UV transillumination.

### 2.4. DNA Sequence Analysis

PCR-positive products were purified with a Roche^®^ purification kit and the obtained amplicons were sequenced using Big Dye Terminator-Applied Biosystem. The nucleotide sequences were aligned with the CLUSTALW program using software BioEdit v7.2.5 (Tom Hall Ibis Biosciences, Carlsbad, CA, USA). The nucleotide sequence identity was compared with reference strains in GenBank database using BLAST (Basic Local Alignment Search Tool) provided by the National Center for Biotechnology Information.

## 3. Results

### 3.1. Tick Collection and Identification

A total of 3286 tick samples were collected. The most frequent source of ticks was the environment, with 2674 (81%) of free-living ticks out of 3286 total.

**Figure 2:**
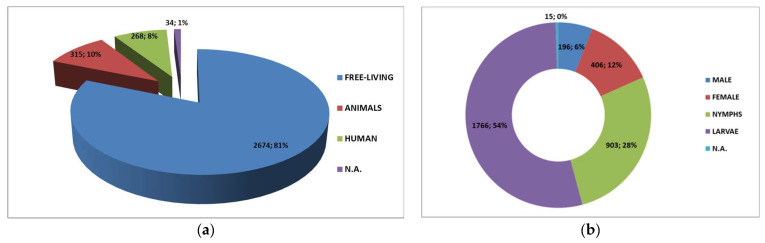
Distribution of source (**a**) and development stages (**b**) of tick samples.

Ticks were collected from the following group of animals: fam. Cervidae, fam. Bovidae, fam. Canidae, fam. Felidae, ord. Rodents, and fam. Suidae. Detailed results of the collection are shown in the Figure 2.

Out of 3286 collected tick samples, 3282 were identified at the species level, while only one was identified at the genus level and three ticks were not determined at the species nor the genus level. Overall, 15 tick species were recognized, the most represented (79.52%) species in the collection was *Ixodes ricinus,* followed by *Rhipicephalus sanguineus* (9.13%), with 11.35% made up of the remaining species. Table 1 summarizes tick ID results.

### 3.2. Rickettsia DNA Detection

All tick samples were screened by PCR for a specific Rickettsia DNA target. The Rickettsia gltA gene was detected in 250 samples.

The overall prevalence of Rickettsia infection was 7.58% (249/3286) of the total samples (Table 2), with a positivity percentage of 6.06%, 20% and 9.33% in ticks collected from environment, animals, and humans, respectively. Table 2 summarizes total results of PCR for Rickettsia in each tick species.

### 3.3. Rickettsia Identification by Sequencing

To confirm results and identify Rickettsia species, all positive PCR products detected in ticks were sequenced. Identification of Rickettsia species based on DNA sequencing was successfully obtained in 200 out of 249 positive samples. *R. monacesis* was the most frequent (56%). Table 3 summarizes data on Rickettsia sequencing in relation to tick species and to the different sources.

## 4. Discussion

The study provides novel insights on the SFG Rickettsiae circulation in the environment in the study area and even if significantly affected by biases, such as the features of the passive monitoring (mainly wild hosts and according seasonality and hunting season) and the low territory coverage of the environmental sampling, it has some strengthening points, such as the diversity of the source of ticks, the length of interval time of the survey, and the consequent high number of samples tested (3286), including 244 from humans. The overall positivity for SFG Rickettsiae was 7.58% (249/3286), which was significantly lower than what was reported in a similar study carried out on ticks collected from wild animals in the Abruzzi region by Pascucci and collaborators [13], who found 52.25% on 178 pools of ixodid ticks, and the results produced by Scarpulla and collaborators [8] on 113 ticks collected from hosts and environment in Latium and Tuscany regions (12.4%), whereas, if we consider only the positivity in ticks collected from animals (19.81%), our results are comparable to the findings of Ebani and collaborators [18] in Tuscany (20.78%). Positivity percentage in our study in free-living ticks (6.06%) was lower than what has been recently reported in a study carried out testing free-living ticks in a suburban area in Latium 25.8% where, however, 255 ticks had been selected randomly out of 518 ticks, and singularly tested [19]. Different ways of pooling ticks in the studies may have affected *Rickettsia* testing results, making them not easily comparable. Ticks belonging to five genera (*Dermacentor*, *Haemaphysalis*, *Hyalomma*, *Ixodes,* and *Rhipicephalus*) were collected during the study, with the most frequent species being *Ixodes ricinus* (79.52%), followed by *Rhipicephalus sanguineus* (9.13%). Both species are documented as the most frequent in central Italy, but even if in our study the proportion is likely affected by the bias represented by the source: 81% of the collection, in fact, was represented by free-living ticks collected by dragging carried out during “spring time”. These sampling techniques and the collection season are, in fact, recognized as the most suitable for *Ixodes ricinus* because of its hunting behavior (ambushing) and for its seasonal dynamic. Similarly, the high prevalence of immaturity in the collection may likely be a consequence of the predominance in free-living ticks and of *Ixodes ricinus* among them. *Ixodes ricinus* was confirmed as the most frequent tick species biting humans in central Italy (244/270), this finding is in accordance to the results of Pascucci and Cammà [20] for the northern part of Marche region. Considering that 9.26% of ticks collected from humans tested positive for *Rickettsia* spp., the human population may be considered at risk for Rickettsiosis in the study area, even though the more detailed epidemiological investigation that comprises spatial analysis needs to be continued. Six different species of *Rickettsia* belonging to the SFG were identified by sequencing—*R. monacensis* was by far the most frequent species in our collection, since more than half of the sequenced samples (56%) belonged to this species. This result is in accordance with the situation previously described in Tuscany [8], and in Emilia Romagna [21]. Similarly, Pascucci and collaborators [13] identified *R. monacensis* in 40.9% out of 93 Rickettsia SFG-positive pools. Remarkably, the frequency of *R. helvetica* in our samples reached 25.37%. Similarly to what was described by Pascucci and collaborators [13], *R. helvetica* was identified exclusively in ticks (51 out of 2613) belonging to *I. ricinus* species, the main vector and natural reservoir [4], while *R. monacensis*, even though found mainly in *I. ricinus* (107 out of 2613), was identified also in *I. acuminatus* and *Rh. sanguineus*, thus confirming what has been suggested by Madeddu and collaborators [22] regarding the possible implication of species other than *I. ricinus* in the transmission of *R. monacensis*. Furthermore, *R. monacensis* and *R. helvetica* had just been detected in *I. ricinus* collected from dogs in the bordering region (Umbria) [23]. Both Rickettsia species are recognized as occasional agents of spotted fever in Italy [1,3,22]. The finding of a Rickettsia identified as *Rickettsia rhipicephali* is in line with what was described by Otranto and collaborator [10] for the first time in Sicily

*R. rhipicephali,* isolated for the first time from *Rh. sanguineus* ticks collected from dogs in Mississippi in 1975 and sporadically reported in Europe [4], was detected in our study, surprisingly not only in *Rhipicephalus* ticks (GenBank Accession Number OL906195) but also in *Ixodes ricinus* (GenBank Accession Numbers OL906194 and OL906196), leading us to hypothesize a possible role in the transmission. This Rickettsia has been identified in ticks from France, Portugal, Greece (including the Island of Cephalonia), Croatia [4], and in 2018 again in Italy using gltA sequencing by Guerden and collaborators [11]. As for other recently identified species, it is not yet confirmed as human pathogen [4]

Conversely, *R. massiliae,* recognized as an agent of human diseases also in Italy, was detected in our study in *Rhipicephalus* ticks, confirming literature data that describe *R. massiliae* presence all over the *Rh. turanicus* and *Rh. sanguineus* geographic distribution [4,13]. Though less common than that found in a similar study carried on in central Italy [13], *R. slovaca* was revealed in our study. This *Rickettsia* is associated with a syndrome in humans characterized by scalp eschars and neck lymphadenopathy following tick bite. Initially, this syndrome was named TIBOLA (tick-borne lymphadenopathy) or DEBONEL (Dermacentor-borne necrotic erythema and lymphadenopathy), and, after 2010, SENLAT (scalp eschar and neck lymphadenopathy after a tick bite) [4] because of its strict but not exclusive association with *Dermacentor* ticks, also described in our study, where *R. slovaca* was also detected in *Hyalomma* and *Ixodes* ticks. The reason for a low frequency of *R. slovaca* may be linked to the low representativeness of *Dermacentor marginatus* ticks in our collection (0.40%). Moreover, in the present study *R. hoogstraalii*, a SFG *Rickettsia* with unknown pathogenicity that is closely related to *R. felis*, an emerging pathogen known to be transmitted by several arthropods [4], was detected in *Ixodes ricinus*. *R. hoogstraalii* had been detected for the first time in Italy by Chisu and collaborators in Sardinia [9]. Tt was originally isolated in 2006, from *H. sulcata* ticks collected from sheep and goats in Croatia [24], in *H. punctata* ticks collected in Cyprus [25], and in *H. punctata* and in *H. sulcata* ticks from Spain [26]. It has spread worldwide and been identified in other tick species [9], and, to our knowledge, this is the second report of *R. hoogstraalii* in Italy and the first in the peninsular territory. Further confirmation of *Rickettsia* species identification should be carried out using different Rickettsia molecular targets, such as OmpA and OmpB. Moreover, the complexity of SFG Rickettsiae biological cycles with different tick species acting as vectors /reservoirs and, consequently, with several vertebrate hosts involved, makes the system extremely sensitive to the influence of climate changes that can be carried out both directly on the vectors modifying its survival and activities, or indirectly on host availability [27]. Thus, additional studies on the relationship between SFG Rickettsiae and climate change should be performed in the study area in order to identify possible scenarios.

## 5. Conclusions

Our findings are the first comprehensive data available on SFG Rickettsiae circulation in the study area and, even if to be supported by further molecular investigation and biases affected, they can be considered as starting point to assess spotted fever risk for humans and to set up surveillance and public awareness activities that are essential prevention tools. A factual assessment of zoonoses risk for humans in contexts placed in natural environments rich in biodiversity with high levels of interaction between humans, domestic and wild animals, vectors, and transmitted pathogens, such as the study area, necessitates a One Health approach that considers human and animal health as strictly interconnected with ecosystem health. This concept is even more essential in the risk assessment for vector-borne diseases.

## Figures and Tables

**Table 1:** Tick ID results.

	Adults		Nymphs	Larvae	N.A.	Total	%
	Male	Female					
*Ixodes ricinus*	116	335	844	1317	1	2613	79.52%
*Rhipicephalus sanguineus*	18	22	12	237	11	300	9.13%
*Haemaphysalis punctata*	41	24	22	106	0	193	5.87%
*Rhipicephalus bursa*	1	2	0	98	0	101	3.07%
*Haemaphysalis parva*	2	5	7	1	0	15	0.46%
*Dermacentor marginatus*	7	6	0	0	0	13	0.40%
*Hyalomma marginatum*	4	8	0	0	0	12	0.37%
*Ixodes acuminatus*	2	0	4	6	0	12	0.37%
*Rhipicephalus pusillus*	0	0	10	0	0	10	0.30%
*Ixodes gibbosus*	0	2	3	0	0	5	0.15%
*Haemaphysalis sulcata*	2	0	1	0	0	3	0.09%
Not determined	0	0	0	0	3	3	0.09%
*Hyalomma lusitanicum*	0	2	0	0	0	2	0.06%
*Ixodes frontalis*	1	1	0	0	0	2	0.06%
*Ixodes* spp.	0	0	0	1	0	1	0.03%
*Ixodes ventalloi*	1	0	0	0	0	1	0.03%
	195	407	903	1766	15	3286	100.00%

**Table 2:** Results of PCR for *Rickettsia* spp.

Tick Species	Free-Living			Animals			Human			N.A.			Total Ticks for Species	Positives for Species	% Positive for Species	% Positive for Total
	Tot	Pos	%	Tot	Pos	%	Tot	Pos	%	Tot	Pos	%				
*Ixodes ricinus*	2176	158	7.26%	192	39	20%	244	23	9.43%	1	0		2613	220	8.42%	6.70%
*Rhipicephalus sanguineus*	260	4	1.54%	28	19	68%	11	1	9.09%	1	1	100%	300	25	8.33%	0.76%
*Haemaphysalis punctata*	123	0		39	0		1	0		30	0		193	0	0.00%	0.00%
*Rhipicephalus bursa*	99	0		2	0		0	0		0	0		101	0	0.00%	0.00%
*Haemaphysalis parva*	4	0		10	1	10%	1	0		0	0		15	1	6.67%	0.03%
*Dermacentor marginatus*	1	0		9	1	11%	3	0		0	0		13	1	7.69%	0.03%
*Hyalomma marginatum*	0	0		11	0		0	0		1	0		12	0	0.00%	0.00%
*Ixodes acuminatus*	9	0		2	1	50%	1	0		0	0		12	1	8.33%	0.03%
*Rhipicephalus pusillus*	0	0		10	0		0	0		0	0		10	0	0.00%	0.00%
*Ixodes gibbosus*	0	0		0	0		5	0		0	0		5	0	0.00%	0.00%
*Haemaphysalis sulcata*	0	0		3	0		0	0		0	0		3	0	0.00%	0.00%
Not determined	0	0		3	0		0	0		0	0		3	0	0.00%	0.00%
*Hyalomma lusitanicum*	0	0		0	0		2	1	50%	0	0		2	1	50.00%	0.03%
*Ixodes frontalis*	2	0		0	0		0	0		0	0		2	0	0.00%	0.00%
*Ixodes spp.*	0	0		0	0		0	0		1	0		1	0	0.00%	0.00%
*Ixodes ventalloi*	0	0		1	0		0	0		0	0		1	0	0.00%	0.00%
TOTAL	2674	162	6.06%	310	62	20.00%	268	25	9.33%	34	1	2.94%	3286	250		7.58%

**Table 3:** *Rickettsia* species identification by sequencing.

		Free-Living		Animals		Human		N.A		Total			
SFG Rickettsia Species Identification in Tick Specimens	Tick Species	Tot	Pos	Tot	Pos	Tot	Pos	Tot	Pos	Tot	Pos	% Positive	Frequence
*R.monacensis*													
	*Ixodes acuminatus*	9	0	2	1	1	0	0	0	12	1	8.33%	
	*Ixodes ricinus*	2176	79	192	17	244	11	1	0	2613	107	4.09%	
	*Rhipicephalus sanguineus*	260	4	28	0	11	0	1	0	300	4	1.33%	
	Total *R. monacensis*										112	3.40%	56.00%
*R.helvetica*	*Ixodes ricinus*	2176	45	192	2	244	4	1	0	2613	51	1.95%	
	Total *R. helvetica*										51	1.55%	25.50%
*R.hoogstraalii*	*Ixodes ricinus*	2176	0	192	2	244	0	1	0	2613	2	0.08%	
	Total *R. hoogstralii*										2	0.06%	1.00%
*R. massiliae*	*Rhipicephalus sanguineus*	260	0	28	9	11	0	1		300	9	3.0%	
	Total *R.massiliae*										9	0.27%	4.50%
*R.rhipicephali*	*Rhipicephalus sanguineus*	260	10	28	0	11	0	1	0	300	10	3.3%	
	*Ixodes ricinus*	2176	9	192	0	244	0	1	0	2613	9	0.34%	
	Total *R. rhipicephali*										19	0.6%	9.50%
*R.slovaca*	*Hyalomma lusitanicum*	0	0	0	0	2	1	0	0	2	1	50%	
	*Ixodes ricinus*	2176	0	192	2	244	0	1	0	2613	2	0.08%	
	*Dermacentor marginatus*	1	0	9	1	3	0	0	0	13	1	7.69%	
	Total *R. slovaca*										4	0.12%	2.00%
*Rickettsia* SFG	*Ixodes ricinus*	2176	1	192	0	244	0	1	0	2613	1	0.04%	
	*Rhipicepahlus sanguineus*	260	0	28	0	11	0	1	1	300	1	0.33%	
	Total *Rickettsia SFG*										2	0.06%	1.00%
*Rickettsia* spp.	*Rhipicephalus sanguineus*	260	0	28	0	11	1	1	0	300	1	0.33%	
	Total *Rickettsia spp.*										1	0.03%	0.50%
Total Positive											200		

## Data Availability

The data presented in this study are available in the present article Pascucci, I.; Antognini, E.; Canonico, C.; Montalbano, M.G.; Necci, A.; Di Donato, A.d.; Moriconi, M.; Morandi, B.; Morganti, G.; Crotti, S.; and Gavaudan S. One Health Approach to Rickettsiosis: A Five Years Study on Spotted Fever Group Rickettsiae in Ticks Collected from Humans, Animals and Environment.
